# Vessel density on optical coherence tomography angiography is prognostic for future disease course in intermediate uveitis

**DOI:** 10.1038/s41598-023-49926-0

**Published:** 2024-02-05

**Authors:** Maximilian W. M. Wintergerst, Nicholas R. Merten, Moritz Berger, Jan H. Terheyden, Lennart J. Overbeck, Matthias Schmid, Frank G. Holz, Robert P. Finger

**Affiliations:** 1 Department of Ophthalmology, University Hospital Bonn, Venusberg-Campus 1, Ernst-Abbe-Straße 2, 53127 Bonn, Germany; 2 Department of Medical Biometry, Informatics and Epidemiology, University Hospital Bonn, Venusberg-Campus 1, 53127 Bonn, Germany; 3 Department of Ophthalmology, University Hospital Mannheim, Theodor-Kutzer-Ufer 1-3, 68167 Mannheim, Germany; 4 Augenzentrum Grischun, KammannEye AG, Chur, Switzerland

**Keywords:** Prognostic markers, Retinal diseases, Uveal diseases

## Abstract

As most rare diseases, intermediate uveitis lacks reliable endpoints necessary for randomized clinical trials. Therefore, we investigated longitudinal changes of retinal and choriocapillaris perfusion on optical coherence tomography angiography (OCT-A) in intermediate uveitis and their prognostic value for future best corrected visual acuity (BCVA) and central retinal thickness (CRT). In this retrospective, longitudinal cohort study eyes of patients with intermediate uveitis were imaged by swept-source OCT-A (macula-centered 3 × 3 mm; PLEX Elite 9000, Zeiss) and stratified into clinically stable, worsened and improved based on changes in clinical parameters. Superficial (SRL) and deep retinal layers (DRL) were automatically analyzed for vessel density (VD) and choriocapillaris layer for non-perfused area (CCNPA) using ImageJ. Mixed-effects regression analysis controlling for age, sex, and OCT-A signal strength index (SSI) was used to evaluate the prognostic value of OCT-A parameters. 91 eyes (62 stable, 12 worsened, and 17 improved) were included in the analysis and mean follow-up time was 296 days. Longitudinal changes of VD were different between all three groups (*p* = 0.002 for SRL and *p* = 0.017 for DRL). Clinically worsened eyes showed a decrease in VD (− 0.032 ± 0.055 for SRL and − 0.027 ± 0.025 for DRL), whereas clinically improved eyes showed an increase in VD (0.037 ± 0.039 for SRL and 0.001 ± 0.023 for DRL). No difference was found for CCNPA. When controlling for age, sex, and SSI, observed differences held true in clinically worsened eyes for DRL (*p* = 0.011) and in clinically improved eyes for SRL (*p* = 0.002). An increase of CCNPA in clinically worsened eyes (*p* = 0.03) compared to clinically stable and improved eyes was evident. Predictive analysis revealed an association of VD in SRL and DRL at baseline with BCVA at follow-up (*p* = 0.039 and *p* = 0.047, respectively) and of VD in SRL at baseline with CRT at follow-up (*p* = 0.046). Alterations in retinal perfusion on OCT-A in intermediate uveitis are partly reversible and OCT-A VD may serve to predict future BCVA and CRT. Thus, perfusion parameters on OCT-A might aid monitoring and serve as prognostic imaging-biomarker.

## Introduction

As with many rare diseases, there is a lack of sufficient evidence from controlled randomized clinical trials regarding the clinical management of intermediate uveitis^1–3^. Quantitative, reliable endpoints are an important prerequisite as they allow both evaluation of potential novel therapies for intermediate uveitis in future clinical trials and clinical management of patients. However, we largely lack such endpoints for uveitis, as inflammation and complications are mostly graded using subjective, relatively unreliable clinical scales^4^. Some trials may even fail to reach their primary endpoint not because of therapeutic inefficacy but because of lack of appropriate structural or functional endpoints^5^. Against this background, there is a need for novel, device-based, quantitative, reproducible endpoints for intermediate uveitis studies^4,5^. This is also reflected in a statement by the European Medicines Agency (EMA) specifically calling for novel endpoints including aspects of structural changes in intermediate uveitis^6^.

Such an endpoint may be based on the microcirculation since reduced vascular density and complexity in superficial as well as deep retinal layers and altered choriocapillaris perfusion have been demonstrated in intermediate uveitis and are associated with functional impairment^7–9^. Thus it is important to assess and monitor even small, sub-clinical changes using state-of-the-art high-resolution retinal imaging such as swept-source optical coherence tomography angiography (OCT-A).

Among other features, involvement of the microvasculature has been implicated in the development of macular edema, a common complication of intermediate uveitis^1^. A breakdown of both the inner and outer blood retina barrier is responsible for macular edema formation^10^. However, impairment of the macular microvasculature may be evident even in the absence of macular edema^7^.

So far, no longitudinal analysis of microvascular changes in intermediate uveitis has been performed and it is unclear whether observed changes of perfusion might be reversible. Furthermore, it is unclear whether OCT-A parameters might be prognostic for clinical outcomes and could, hence, serve as prognostic factors.

The aim of this study was to evaluate changes in perfusion on OCT-A in intermediate uveitis over time, their permanence as well as associations with clinical outcomes during longitudinal review.

## Methods

### Subjects and clinical examination

Diagnosis of intermediate uveitis was based on the diagnostic criteria of the SUN working group^11^. In this retrospective study, patients with at least one follow-up examination were included from the uveitis outpatient clinic at the Department of Ophthalmology, University Hospital Bonn, Germany. Ethical approval was obtained from the ethics committee of the University of Bonn and waived informed consent was approved (approval ID 548/20, Ethik-Kommission—Medizinische Fakultät Bonn). The study adhered to the Declaration of Helsinki. Eyes were excluded, if no OCT-A acquisition was possible (e.g. due to optical media opacities). Other exclusion criteria were reduced OCT-A image quality (shadowing or blurred en face OCT-A image), diabetes mellitus or any other retinal disease except for intermediate uveitis.

Clinical and demographic data were obtained from the medical charts. Best-corrected visual acuity (BCVA) was converted into logMAR. To control for a possible confounding effect of axial length on retinal / choriocapillary perfusion^12^, spherical refractive error was included as a surrogate in the analysis, as axial length measurements were not available in this retrospective cohort. Inflammation was graded clinically by assessment of anterior chamber cells (based on the SUN working group grading scale^11^), vitreous haze (based on the proposed scale for vitreous haze grading in uveitis^13^), vitreous cells (based on the proposed grading scale by Nussenblatt et al.^14^), snowballs, snowbanks, and presence of vasculitis.

### Image acquisition and analysis

Study participants were imaged with swept-source OCT-A with 100k A-scans/second at 1060 nm (Zeiss PLEX Elite 9000, Carl Zeiss Meditec, Dublin, California, USA) using a 3 × 3 mm scan pattern of the macula, formed by 300 horizontal A-lines at 300 vertical locations. Each A-line was acquired over a depth of 3 mm and contained 1536 pixels. In case of minor focal media opacities the alignment of the OCT-A optical beam projection entering the eye was slightly altered to circumvent opacities and acquire an image without shadowing. The OCT-A images were generated using an optical microangiography complex (OMAGc) algorithm^15^. A general sliding slab method was used to process the 3-dimensional OCT-A data for the purpose of removing decorrelation tail artifacts within the OCT-A volume (see Bagherinia et al.^15^ and Wintergerst et al.^7^ for further details). Using the proprietary algorithm from the OCT-A device, OCT-A en face images of the superficial (spanning the nerve fiber, ganglion cell, and inner plexiform layers) and deep retinal layers (spanning the inner nuclear and outer plexiform plus Henle fiber layers) and choriocapillaris layers (4–20 µm below Bruchs’ membrane segmentation^16^) layers were generated using the maximum projection of each particular slab within the artifact-corrected volume. Study participants were also imaged with conventional optical coherence tomography (OCT) (Heidelberg Spectralis, Heidelberg Engineering, Heidelberg, Germany) for central retinal thickness (CRT) within a 0.5 mm radius around the fovea (ETDRS central 1 mm circle) and presence and severity level of macular edema. Macular edema was classified as absent, present without change of foveal contour (only very mild intraretinal fluid with still intact foveal contour) and present with change of foveal contour.

Quantification of the OCT-A images was performed with Fiji (an expanded version of ImageJ version 1.51a, National Institutes of Health, Bethesda, MD, USA)^17^. En face images of the superficial and deep retinal layer were binarized with automatic thresholding algorithms and vessel density (VD) of superficial and deep retinal layers were calculated as previously described^18^. Briefly, the “Li” algorithm^19^ was used for binarization of superficial layers and the “Moments” algorithm^20^ for deep layers. Choriocapillaris non-perfused area (CCNPA) was analyzed using the Phansalkar method (with a radius of 5 pixels) as previously described^21^. Projection artifacts from superficial retinal vessels^22^ were removed for analysis of deep retinal and choriocapillary layers using the proprietary algorithm provided by the OCT-A device (Zeiss PLEX Elite 9000, Carl Zeiss Meditec, Dublin, California, USA). The analysis in this study was focused on VD as retinal OCT-A outcome measure, since this is the most basic and relevant OCT-A parameter.

### Clinical grading and statistical analyses

Change of macular edema classification (see above), change of CRT by 100 μm or more, change of vitreous cells, vitreous haze or anterior chamber cells by two levels or more, or change in terms of presence / absence of snowballs, snowbanks, or vasculitis were considered relevant clinical changes. Patients were divided into the following three groups based on these relevant clinical changes between baseline and follow-up: clinically ‘worsened’, ‘stable’, and ‘improved’. If more than two examinations were available, the two examinations with the most pronounced clinical change were selected in the clinically worsened and improved group. In the clinically stable group, examinations with least clinical change were selected for statistical analysis. Statistical analyses were performed with R (R: A Language and Environment for Statistical Computing, version 4.0.3, R Core Team, R Foundation for Statistical Computing, Vienna, Austria, 2020). Friedman test for repeated measures was used for the comparison of OCT-A parameter changes between the three clinical groups unless otherwise indicated. *P*-values < 0.05 were considered statistically significant. The comparison of OCT-A parameter changes and clinical groups was additionally adjusted for age, sex and signal strength index (SSI) at baseline and follow up as possible confounders using a multivariable linear mixed-effects regression model (including a random intercept for each patient).

Furthermore, multivariable linear mixed-effects regression analyses using all available visits (as defined in the previous paragraph) were performed in order to evaluate the prognostic value of OCT-A parameters for BCVA and CRT. Future BCVA and future CRT were defined as dependent and current OCT-A parameters, current OCT-A SSI and current BCVA and CRT as independent variables. If there were more than two examinations with sufficient image quality available for one eye, the subsequent examination was defined as future and the previous as current examination, e.g. the second examination was associated to the third examination, the third examination was associated to the fourth, etc.

## Results

### Demographics and clinical characteristics

Twenty eyes were excluded due to insufficient OCT-A image quality. The remaining 91 eyes (52 patients) were included and divided into the three groups as defined above. The clinically stable group included 62 eyes, the clinically worsened group 12 eyes and the clinically improved group 17. Baseline characteristics of the sample are reported in Table 1. There was no statistically significant difference in spherical refractive error between the three groups.

**Table 1:** Baseline characteristics of the sample.

	Mean ± SD (range) or n (%)					
	Clinically worsened group		Clinically stable group		Clinically improved group	
	(n = 12 eyes)		(n = 62 eyes)		(n = 17 eyes)	
	Baseline	Follow-up	Baseline	Follow-up	Baseline	Follow-up
Age at baseline (years)	48 ± 18 (26–80)		47 ± 18 (17–90)		41 ± 21 (17–90)	
Sex (male)	7 (58%)		25 (40%)		12 (71%)	
Spherical refractive error (dpt)	0.10 ± 1.11		0.76 ± 2.34		− 0.06 ± 1.77	
Best corrected visual acuity (LogMAR)	0.25 ± 0.23	0.44 ± 0.31	0.06 ± 0.18	0.07 ± 0.18	0.32 ± 0.25	0.05 ± 0.08
Central retinal thickness (µm)	278 ± 68	398 ± 166	300 ± 59	299 ± 54	448 ± 117	296 ± 30
Lens status (phakic)	8 (67%)	5 (42%)	47 (76%)	47 (76%)	13 (76%)	12 (71%)
Macular edema						
No macular edema	10 (83%)	2 (17%)	56 (90%)	57 (92%)	0 (0%)	16 (94%)
Macular edema without change of macular contour	2 (17%)	1 (8%)	4 (7%)	3 (5%)	8 (47%)	1 (6%)
Macular edema with change of macular contour	0 (0%)	9 (75%)	2 (3%)	2 (3%)	9 (53%)	0 (0%)
OCT-A signal strength index	8.83 ± 0.83	8.17 ± 1.11	9.53 ± 0.59	9.41 ± 0.67	8.82 ± 1.07	9.41 ± 0.71
Duration of uveitis at baseline (months)	85 ± 57		99 ± 64		73 ± 55	
Time between baseline and follow-up (days)	327 ± 265		264 ± 229		388 ± 326	

dpt = diopters; SD = standard deviation; OCT-A = optical coherence tomography angiography.

### Longitudinal changes of OCT-A parameters

**Figure 1:**
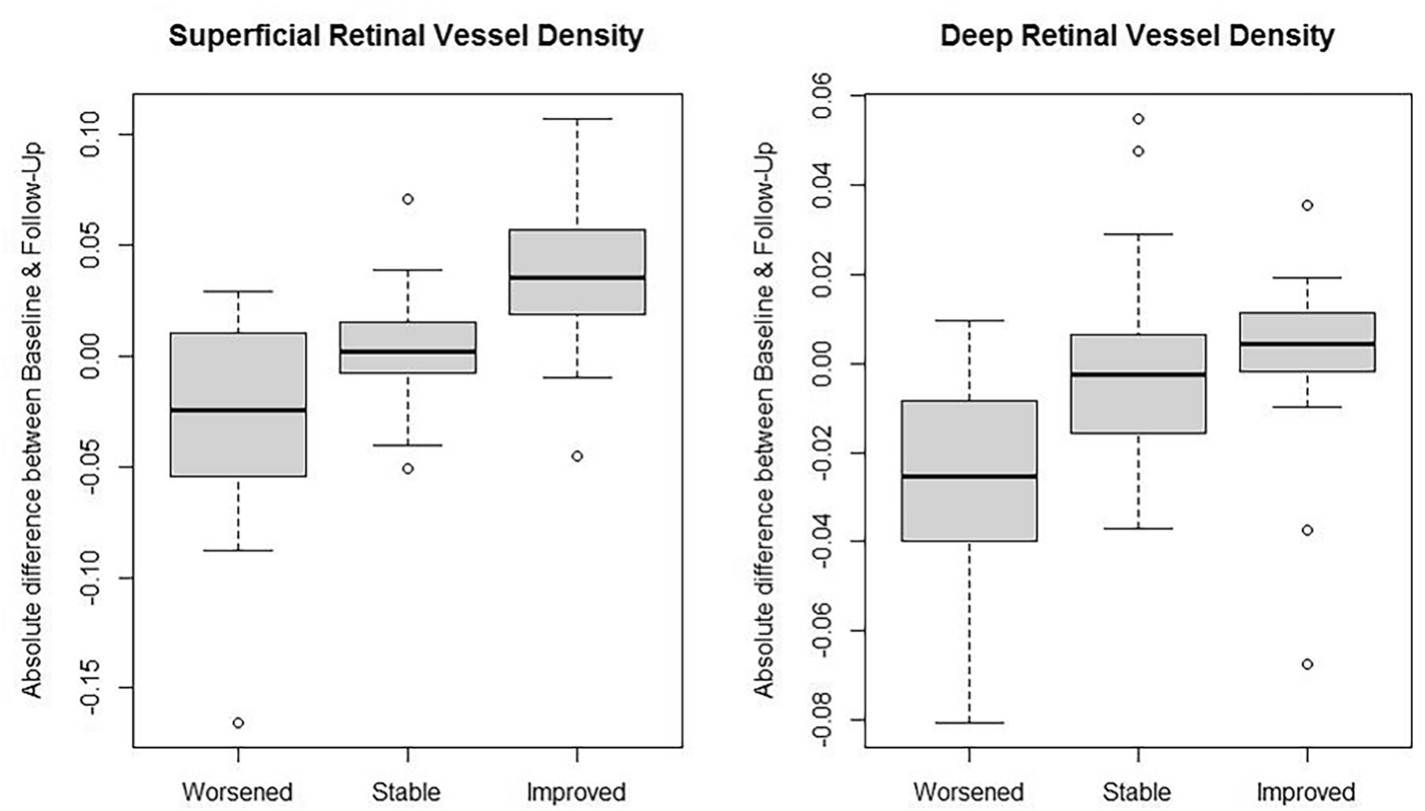
Change in vessel density of superficial and deep retinal layers. Analysis of absolute difference in superficial and deep retinal layer vessel density between baseline and follow-up on optical coherence tomography angiography in eyes with intermediate uveitis. Change in vessel density is compared between the three groups “worsened”, “stable”, and “improved”, defined by their clinical development. Outliers were defined as values over 1.5 interquartile range below the first quartile or above the third quartile.

**Figure 2:**
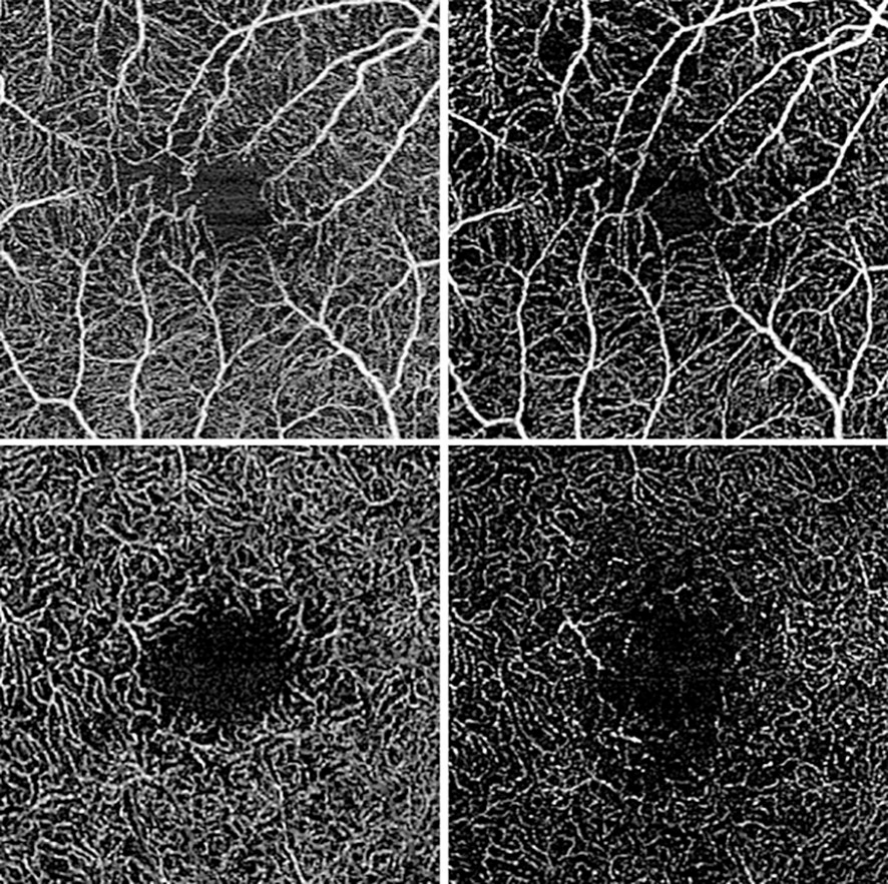
Exemplary OCT-A en face images of clinically worsened eyes. Vessel density of both superficial (top row) as well as deep (bottom row) retinal layers decreases in clinically worsened eyes (left: baseline; right: follow-up). Images were cropped for depicting exactly the same region of interest. No additional postprocessing was applied after export from the OCT-A device.

**Figure 3:**
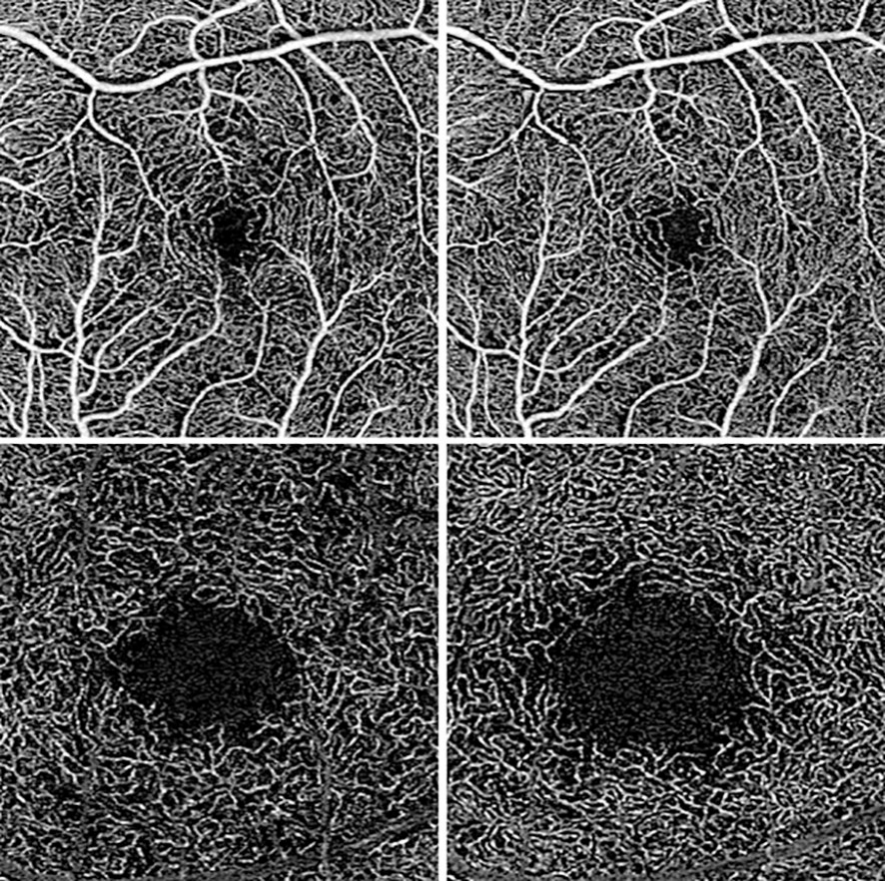
Exemplary OCT-A en face images of clinically improved eyes. Vessel density of both superficial (top row) as well as deep (bottom row) retinal layers increases in clinically improved eyes (left: baseline; right: follow-up). Images were cropped for depicting exactly the same region of interest. No additional postprocessing was applied after export from the OCT-A device.

Comparisons of change in VD between the three clinical groups showed significant differences for both the superficial and deep retinal layers (Fig. 1, *p* = 0.002 for superficial and *p* = 0.017 for deep retinal layers). There was no relevant change in VD in the clinically stable group (0.003 ± 0.021 for SRL and − 0.004 ± 0.017 for DRL), whereas VD decreased in the clinically worsened (− 0.032 ± 0.055 for SRL and − 0.027 ± 0.025 for DRL, Fig. 2) and increased in the clinically improved group (0.037 ± 0.039 for SRL and 0.001 ± 0.023 for DRL, Fig. 3). There was no significant difference of CCNPA between clinically worsened and stable group (0.097 ± 0.088, 0.026 ± 0.058, and − 0.004 ± 0.099 for worsened, stable, and improved group, respectively, supplement figure, *p* = 0.20).

SSI was associated with superficial retinal layer VD at baseline and follow-up, and with deep retinal layer VD at follow-up. Observed differences between clinically stable and worsened groups in deep retinal layers and between clinically stable and improved groups in superficial retinal layers held true in multivariable regression analyses. In confounding analyses, an increase of CCNPA was revealed in clinically worsened eyes compared to clinically stable and improved eyes (*p* = 0.03).

### Evaluation of the prognostic value of OCT-A parameters

In total, 304 examinations of 91 eyes were included in the multivariable regression analyses. Both current VD in superficial and deep retinal layers showed a significant association with future BCVA (Table 2) and current VD in superficial retinal layers additionally showed a significant association with future CRT (Table 3). CCNPA was not significantly associated with future BCVA or CRT (*p* = 0.298 and *p* = 0.864, respectively).

**Table 2:** Prognostic analyses of OCT-A parameters for future BCVA.

	Model 1 with superficial retinal vessel density as OCT-A parameter			Model 2 with deep retinal vessel density as OCT-A parameter			Model 3 with choriocapillaris non-perfused area as OCT-A parameter		
	Estimate	Std. Error	*p*	Estimate	Std. Error	*p*	Estimate	Std. Error	*p*
Intercept	3.2 × 10^–1^	1.2 × 10^–1^	**0.010**	3.3 × 10^–1^	1.2 × 10^–1^	**0.008**	1.9 × 10^–1^	1.3 × 10^–1^	0.135
Association of OCT-A parameter with future BCVA (LogMAR)	− 5.9 × 10^–1^	2.8 × 10^–1^	**0.039**	− 8.1 × 10^–1^	4.0 × 10^–1^	**0.047**	1.4 × 10^–1^	1.3 × 10^–1^	0.298
Current OCT-A SSI	− 1.4 × 10^–2^	1.3 × 10^–2^	0.283	− 2.3 × 10^–2^	1.2 × 10^–2^	0.064	− 2.0 × 10^–2^	1.3 × 10^–2^	0.114
Current BCVA (LogMAR)	6.6 × 10^–1^	5.3 × 10^–2^	**< 0.0001**	7.0 × 10^–1^	4.9 × 10^–2^	**< 0.0001**	7.3 × 10^–1^	4.6 × 10^–2^	**< 0.0001**

Multivariable regression analyses using linear mixed models for future BCVA as dependent variable including a random intercept for each patient.

BCVA = best corrected visual acuity; SSI = signal strength index; dependent variables: future BCVA; independent variables: current BCVA, current OCT-A SSI, current superficial/deep retinal vessel density/choriocapillaris non-perfused area; the estimate, standard error, and *p*-value for the respective OCT-A parameter are displayed in row titled “association of OCT-A parameter with future BCVA (LogMAR)”.

Significant values are in bold.

**Table 3:** Prognostic analyses of OCT-A parameters for future CRT.

	Model 4 with superficial retinal vessel density as OCT-A parameter			Model 5 with deep retinal vessel density as OCT-A parameter			Model 6 with choriocapillaris non-perfused area as OCT-A parameter		
	Estimate	Std. error	*p*	Estimate	Std. error	*p*	Estimate	Std. error	*p*
Intercept	3.0 × 10^2^	7.5 × 10^1^	**< .0001**	2.5 × 10^2^	7.5 × 10^1^	**0.001**	2.4 × 10^2^	7.4 × 10^1^	**0.001**
Association of OCT-A parameter with future CRT (OCT)	− 3.1 × 10^2^	1.6 × 10^2^	**0.046**	− 1.0 × 10^2^	2.4 × 10^2^	0.664	1.3 × 10^1^	8.0 × 10^1^	0.864
Current OCT-A SSI	3.7 × 10^0^	7.0 × 10^0^	0.595	− 4.5 × 10^–1^	6.7 × 10^0^	0.946	− 4.0 × 10^–1^	6.9 × 10^0^	0.954
Current CRT (OCT)	2.0 × 10^–1^	6.9 × 10^–2^	**0.004**	2.3 × 10^–1^	6.8 × 10^–2^	**0.001**	2.4 × 10^–1^	6.8 × 10^–2^	**0.0005**

Multivariable regression analyses using linear mixed models for future CRT as dependent variable including a random intercept for each patient.

CRT = central retinal thickness; OCT = optical coherence tomography; SSI = signal strength index; dependent variable: future CRT; independent variables: current CRT, current OCT-A SSI, current superficial/deep retinal vessel density/choriocapillaris non-perfused area; the estimate, standard error, and *p*-value for the respective OCT-A parameter are displayed in row titled “association of OCT-A parameter with future CRT (OCT)”.

Significant values are in bold.

## Discussion

Our findings indicate that changes of VD in superficial and deep retinal layers in intermediate uveitis are reversible and associated with clinical disease course. Furthermore OCT-A parameters were prognostic for future BCVA and CRT. Hence, OCT-A may allow for the identification of patients at risk for future disease progression and could, therefore, be considered to be implemented in routine clinical monitoring. Furthermore, OCT-A parameters might serve as structural endpoints for future randomized controlled clinical trials.

To the best of our knowledge, this is the first longitudinal study on the application of OCT-A in intermediate uveitis. Cross-sectional previous studies revealed a reduction of microvascular perfusion and complexity in superficial and deep retinal layers and the choriocapillaris in eyes with intermediate uveitis compared to healthy controls^7–9^. The role of macular edema in this context has been controversially discussed^9,23,24^. Also in this study, displacement of retinal vessels by intraretinal cysts in macular edema is likely a relevant cause for impaired retinal perfusion, as displacement might artificially alter retinal perfusion parameters on OCT-A. However, as previously reported, reduced perfusion is also present in the absence of macular edema^7,23^.

Our study confirms the association of OCT-A parameters in superficial and deep retinal layers and the choriocapillaris with BCVA and CRT described in previous studies. Due to the cross-sectional nature, previous studies could, however, not assess whether observed perfusion changes were reversible^7^. Our longitudinal study found an increase of retinal perfusion in clinically improved eyes and a decrease in clinically worsened eyes, therefore, observed alterations in retinal perfusion are—at least partially—reversible. Furthermore, we detected a statistically significant increase of CCNPA in clinically worsened compared to clinically stable eyes.

Leukocyte adherence to vessel walls (leukostasis) and leukocyte extravasation are of considerable importance in the pathophysiology of uveitis and contribute to vascular leakage^25–29^. As leukostasis has been hypothesized as an explanation for reversible impairment of microvascular perfusion in other retinal diseases before^30–34^, we speculate, that leukostasis may contribute to the observed reversible impairment of microvascular perfusion in intermediate uveitis. A role of leukostasis in intermediate uveitis is further supported by evidence of treatment effects by drugs which target leukocyte trafficking such as Natalizumab and Fingolimod in certain intermediate uveitis subtypes^35^.

Longitudinal studies of posterior uveitis (acute posterior multifocal placoid pigment epitheliopathy and syphilitic posterior placoid chorioretinitis) indicated reversible impairment of choriocapillaris perfusion^36,37^. On the contrary, in our study of intermediate uveitis, impairment of choriocapillary perfusion was irreversible. This might be related to a greater inflammatory damage in the choriocapillaris vasculature in intermediate uveitis. However, exact causes remain to be clarified.

Superficial and deep retinal VD were prognostic for future BCVA in our study. Likewise, superficial retinal VD was prognostic for future CRT. Especially the perfusion of the inner nuclear layer's outer boundary is crucial for photoreceptor, horizontal and bipolar cell function^38^, which might contribute to this finding. In other retinal diseases, including age-related macular degeneration, several OCT-A parameters were identified as prognostic imaging biomarkers for future disease progression^39^. Thus, monitoring OCT-A changes in intermediate uveitis may aid in better determining individual prognosis.

Limitations of our study include a relatively small sample size of the clinically worsened and improved group, however this reflects real-world settings. This limitation only applies to the comparison of clinical groups, as the complete available data was used for the predictive analysis. Furthermore, this is a retrospective study and we did not analyze the effect of recurrent episodes of intraocular inflammation. Strengths include the prospective nature of our study and the large dataset used for our predictive analysis. Furthermore, we controlled for possible confounders using regression analyses, standardized OCT-A imaging, image analysis, and uveitis phenotyping based on the SUN criteria.

In conclusion, impairment of retinal perfusion in intermediate uveitis was reversible and OCT-A parameters were prognostic for future BCVA and CRT. Hence, OCT-A may aid in monitoring intermediate uveitis and provide additional information for better assessment of prognosis. Future studies should validate its usefulness as an endpoint in prospective longitudinal trials.

### Supplementary Information

Supplementary Figure.

Supplementary Table.

## Data Availability

The datasets generated and analyzed during the current study are not publicly available due to data protection regulations but are available from the corresponding author on reasonable request.
