# Supplementary material for: Vessel density on optical coherence tomography angiography is prognostic for future disease course in intermediate uveitis
Source: Sci Rep. 2024 Feb 5;14:2933. doi: 10.1038/s41598-023-49926-0 (PMC10844199; doi:10.1038/s41598-023-49926-0)
Supplement: Supplementary file 1 — Supplementary Figure. [file 41598_2023_49926_MOESM1_ESM.docx]

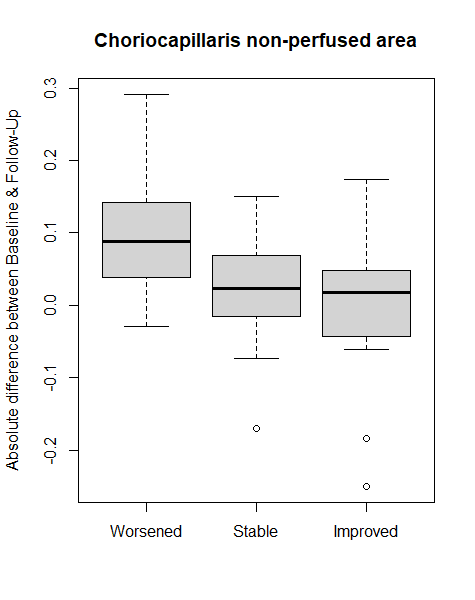


**Supplemental Figure.** Choriocapillaris non-perfused area

Longitudinal quantitative analysis of optical coherence tomography angiography parameters of choriocapillaris in eyes with intermediate uveitis. Choriocapillaris non-perfused area is presented for each of the three groups characterized by their clinical development. Outliers were defined as values over 1.5 interquartile range below the first quartile or above the third quartile.
