# Supplementary material for: Vessel density on optical coherence tomography angiography is prognostic for future disease course in intermediate uveitis
Source: Sci Rep. 2024 Feb 5;14:2933. doi: 10.1038/s41598-023-49926-0 (PMC10844199; doi:10.1038/s41598-023-49926-0)
Supplement: Supplementary file 2 — Supplementary Table. [file 41598_2023_49926_MOESM2_ESM.docx]

**Supplemental Table.** Confounding Analysis

Multivariable regression analysis using linear mixed-effects models including a random intercept for each patient (worse and better: compared to stable group)

|  |  | | | Change in… | | |  | | |
| --- | --- | --- | --- | --- | --- | --- | --- | --- | --- |
|  | Superficial retinal vessel density | | | Deep retinal vessel density | | | Choriocapillaris non-perfused area | | |
|  | Estimate | Std. Error | p | Estimate | Std. Error | p | Estimate | Std. Error | p |
| Intercept | -6.1 x 10^-2^ | 4.7 x 10^-2^ | .197 | -1.2 x 10^-2^ | 3.4 x 10^-2^ | .737 | -1.8 x 10^-2^ | 1.1 x 10^-1^ | .864 |
| Clinically worsened | -1.9 x 10^-2^ | 1.1 x 10^-2^ | .073 | -2.0 x 10^-2^ | 7.7 x 10^-3^ | **.011** | 5.1 x 10^-2^ | 2.4 x 10^-2^ | **.033** |
| Clinically improved | 2.4 x 10^-2^ | 7.7 x 10^-3^ | **.002** | 1.1 x 10^-3^ | 5.6 x 10^-3^ | .850 | -5.7 x 10^-3^ | 1.8 x 10^-2^ | .749 |
| Age | 2.3 x 10^-4^ | 1.9 x 10^-4^ | .224 | 3.1 x 10^-5^ | 1.3 x 10^-4^ | .815 | 2.3 x 10^-4^ | 4.0 x 10^-4^ | .560 |
| Sex | -1.2 x 10^-2^ | 6.9 x 10^-3^ | .082 | 5.9 x 10^-4^ | 5.0 x 10^-3^ | .906 | -2.2 x 10^-2^ | 1.5 x 10^-2^ | .147 |
| OCT-A SSI at BL | -1.2 x 10^-2^ | 4.1 x 10^-3^ | **.004** | -5.2 x 10^-3^ | 3.0 x 10^-3^ | .079 | 4.1 x 10^-2^ | 9.3 x 10^-3^ | **< .0001** |
| OCT-A SSI at FU | -1.8 x 10^-2^ | 4.0 x 10^-3^ | **< .0001** | 5.8 x 10^-3^ | 2.9 x 10^-3^ | **.045** | -3.7 x 10^-2^ | 9.2 x 10^-3^ | **< .0001** |
| SSI = signal strength index; BL = Baseline; FU= Follow-up | | | | | | | | | |
